# Clinical analysis and prognosis of synchronous and metachronous multiple primary malignant tumors

**DOI:** 10.1097/MD.0000000000006799

**Published:** 2017-04-28

**Authors:** Meng Lv, Xiao Zhang, Yanwei Shen, Fan Wang, Jiao Yang, Biyuan Wang, Zheling Chen, Pan Li, Xiaoman Zhang, Shuting Li, Jin Yang

**Affiliations:** a Department of Oncology, The First Affiliated Hospital of Xian Jiaotong University, Xi’an; b Department of Medical Oncology, Xianyang Center Hospital, Xianyang, Shaanxi, PR China.

**Keywords:** clinical characteristics, metachronous, multiple primary malignant tumors, prognosis, synchronous

## Abstract

The aim of this study was to determine the clinical features, treatment factors, and prognosis of patients with multiple primary malignant tumors (MPMTs). In total, 161 patients with MPMTs at our hospital (The First Affiliated Hospital of Xi’an Jiaotong University, Xi’an, Shaanxi, China) were analyzed in this study. We found that among 161 patients with MPMTs, 78 (48.4%) patients had synchronous tumors and 83 (51.6%) patients had metachronous tumors. Most clinical and pathological features were similar in both groups. Most patients with MPMTs were men and older patients (>50 years old), and adenocarcinoma was the most frequent pathology type. The most frequent location of all MPMTs was the digestive system. The leading tumor association was between digestive–digestive tumors, also. However, patients with synchronous tumors and MPMTs of the digestive system showed a shorter survival time. In the metachronous cancer group, the median interval time was 60 months, and a short interval time (≤60 months) was associated with a shorter survival time. In addition, survival time was increased in the younger age group (≤50 years old) and in patients who accepted surgery-based comprehensive therapy. However, only interval time (≤60 months) was an independent prognostic factor associated with survival for the metachronous cancer group. Therefore, careful surveillance and follow-up are especially important in these patients.

## 1 Introduction

In the 21st century, cancer has become a serious hazard to human health. It is reported that there were about 4292,000 newly diagnosed cancer cases in 2015 in China, corresponding to almost 12,000 new cancer diagnoses on average each day.^[1]^ In the United States, a total of 1658,370 new cancer cases are projected to occur in 2015.^[2]^ However, with the development of modern diagnostic procedures and chemotherapy/radiation/target therapy, the survival rate is increasing. This allows more patients with cancer to survive long enough to develop multiple primary malignant tumors (MPMTs).^[3]^

MPMTs were first described by Billroth^[4]^ in 1889 and reported in a detailed study by Warren and Gates^[5]^ in 1932. Based on criteria proposed by Warren and Gates, diagnosis of MPMTs was dependent on each tumor must have clear evidence of malignancy on histologic examination, each tumor must be geographically separate and distinct, and the possibility of a metastatic lesion having spread from a prior cancer must be excluded. International rules for MPMTs are more detailed, and tumors arising in an organ or a pair of organs or a tissue are usually considered to be 1 tumor. However, there are 2 exceptions to this rule: systemic cancers potentially involving many different organs should only be counted once in any individual, and cancers with different histology should be regarded as multiple cancers, even if they are diagnosed simultaneously at the same site.^[6,7]^

The incidence of MPMTs has been reported to range from 0.52% to 11.7% in various studies from different countries.^[8–10]^ In different geographical regions, the incidence, characteristics, and survival rates associated with MPMTs have been found to be diverse. Further studies are needed to help understand this disease.

In this study, we analyzed the clinical characteristics and prognosis of patients with MPMTs in our hospital in northwest China from January 2008 to February 2015. We also examined the risk factors associated with poor prognosis for patients with MPMTs.

## 2 Patients and methods

### 2.1 Data collection

A total of 161 patients at our hospital (The First Affiliated Hospital of Xi’an Jiaotong University, Xi’an, Shaanxi, China) with a diagnosis of MPMTs between January 2008 and February 2015 were reviewed according to the criteria proposed by Warren and Gates.^[5]^

MPMTs may be defined as synchronous or metachronous tumors. “Synchronous” tumors refer to cases in which the second primary cancer is diagnosed within 6 months of the primary cancer; “metachronous” tumors refer to cases in which the second primary cancer is diagnosed more than 6 months after the diagnosis of the first primary cancer.

All pathological results were confirmed by the Pathology Department of our hospital. Records containing uncertain data, such as indecisive pathologic reports or absent registration of former tumors, were excluded. Six tumors with ≥3 primary tumors were also excluded from the study.

This study was reviewed and approved by the Human Ethics Committee of The First Affiliated Hospital, College of Medicine of Xi’an Jiaotong University.

### 2.2 Treatment and overall survival

Patients with MPMTs were treated with surgery, chemotherapy, radiotherapy, and comprehensive treatment according to clinical guidelines. Overall survival (OS) was calculated as the number of months between the date of diagnosis and the date of death or the date of the end of the follow-up (January 2016).

### 2.3 Statistical analysis

For statistical analysis, the SPSS (version 17.0; SPSS Inc., Chicago, IL) program was used. Differences between groups were evaluated by the chi-square or fisher exact test. Survival probabilities were estimated using the Kaplan–Meier method. Cox proportional hazard multivariate analysis was performed to identify independent factors associated with death. All *P* values <.05 were considered statistically significant.

## 3 Results

### 3.1 Clinical features of MPMT patients

In total, 15,683 patients were diagnosed with malignant tumors in our hospital between January 2008 and February 2015. Of these, 161 (1.0%) patients were diagnosed with MPMTs. Of these 161 patients, 78 (48.4%) had 2 synchronous tumors, and 83 (51.6%) patients had 2 metachronous tumors (Table 1).

**Table 1:**
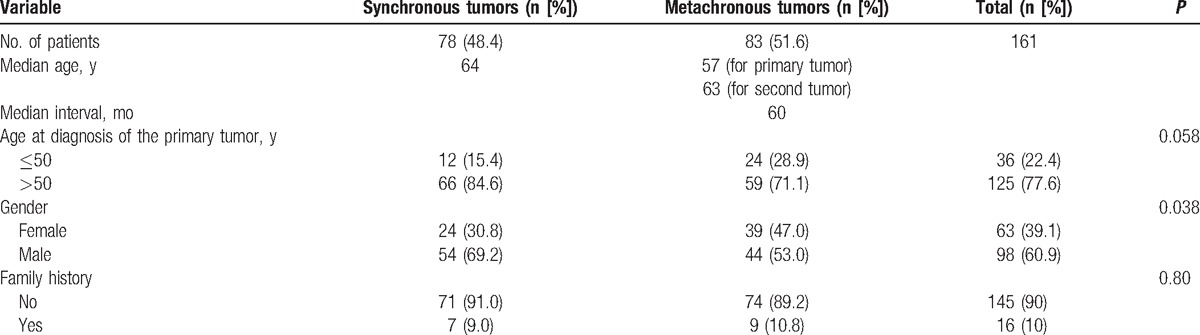
Clinical characteristics.

In the synchronous cancer group, the median age was 64 years. In the metachronous cancer group, the median age was 57 years at the time of diagnosis of the first primary cancer and 63 years at the time of diagnosis of the second primary cancer. The interval time (the time between the date of diagnosis of the first primary cancer and the date of diagnosis of the second primary cancer) was evaluated only for metachronous tumors. The median interval for metachronous cancers was 60 months (range, 7–360 months, Table 1). Our results showed an interval of within 60 months for 57.8% (48/83) of patients with metachronous cancers. Breast cancer and urogenital system cancer were the most common first primary cancers in patients showing a long interval time (≥120 months). In both the synchronous and metachronous cancer groups, most patients were over 50 years old (84.6% and 71.7%, respectively). However, there were more patients in the metachronous cancer group of less than 50 years of age than in the synchronous cancer group (28.9% vs 15.4%), indicating that patients with metachronous primary cancer were generally younger.

In total, 63 (39.1%) patients with MPMTs were females and 98 (60.9%) were males. In both the synchronous and metachronous cancer groups, men were more frequent, and there was a statistical difference in the distribution of synchronous and metachronous cancer cases between gender groups (*P* = .038; Table 1).

The percentage of patients who had a family history of cancer was similar between the synchronous and metachronous cancer groups (9.0% vs 10.8%, Table 1).

### 3.2 Pathological features of MPMTs

Among all of the MPMT cases analyzed, the frequent pathology types were adenocarcinomas (49.3%), squamous carcinoma (26.1%), hematopoietic and lymphoid tissues (8.1%), transitional cell carcinoma (6.2%), and sarcomas and soft tissue tumors (0.9%, Table 2). In addition, other specific carcinomas (9.3%) in Table 2 include neuroendocrine tumors, gastrointestinal stromal tumors (GISTs), embryonal carcinoma, malignant melanoma, seminomas, renal clear cell carcinoma, and so on. Among synchronous tumors specifically, the most frequent pathology groups were adenocarcinomas (55.1%) and squamous carcinomas (23.1%). In the first metachronous tumor and the second metachronous tumor groups, the most frequent pathology types were also adenocarcinomas (42.2% and 45.8%, respectively) and squamous carcinomas (2.5% and 25.3%, respectively, Table 2). Adenocarcinomas were therefore the most common pathological type in both the synchronous and metachronous cancer groups.

**Table 2:**
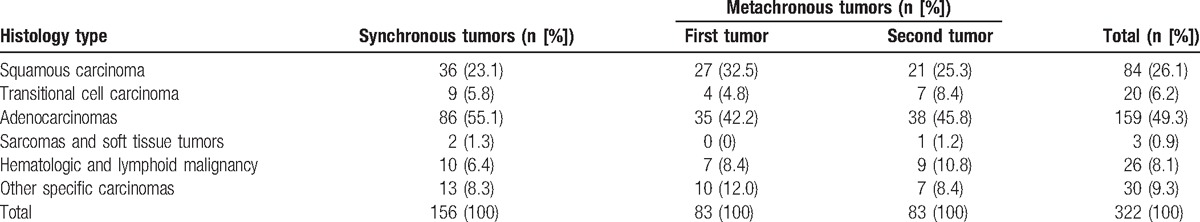
Pathologic characteristics.

### 3.3 Distribution of MPMTs

Tumors of the digestive system were the most common MPMTs (39.1%), followed by urogenital system (24.5%) and respiratory system (17.1%) tumors (Table 3). In the synchronous tumor group, the top 3 systems were the digestive (48.7%), urogenital (21.8%), and respiratory (15.4%) systems. In the first metachronous tumor group, the most commonly affected area was the urogenital system (32.5%), followed by the digestive system (27.7%) and breast tissue (15.6%), whereas in the second metachronous tumor group, the digestive system (32.5%), respiratory system (25.3%), and urogenital system (21.7%) were most commonly affected (Table 3).

**Table 3:**
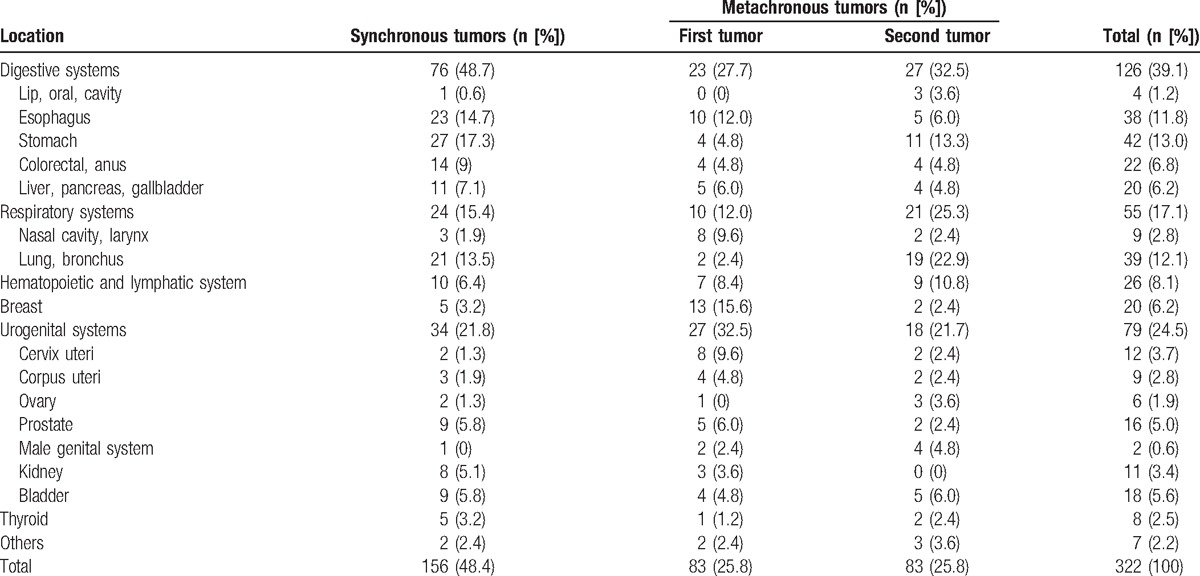
The distribution of synchronous and metachronous tumors.

The most frequent sites for tumor localization in all cases of MPMTs were the stomach (13%), lung (12%), and esophagus (11.8%, Table 3). Specifically, the most frequent tumor localization of the synchronous tumors was stomach (17.3%), whereas the leading localization of the first and second metachronous tumor was breast (15.6%) and lung (22.9%), respectively (Table 3).

Among males, tumors were most frequently located in the digestive (48.5%), respiratory (20.9%), and urogenital (20.9%) system. Among females, the urogenital system (30.2%), digestive system (24.6%), and breast tissue (15.9%) were the predominant sites for tumor localization (Table 4).

**Table 4:**
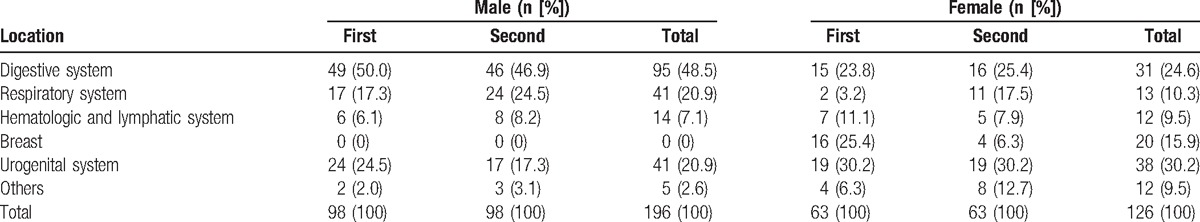
The distribution of MPMTs in male and female patients.

### 3.4 The associations between different systems with regard to MPMTs

**Figure 1:**
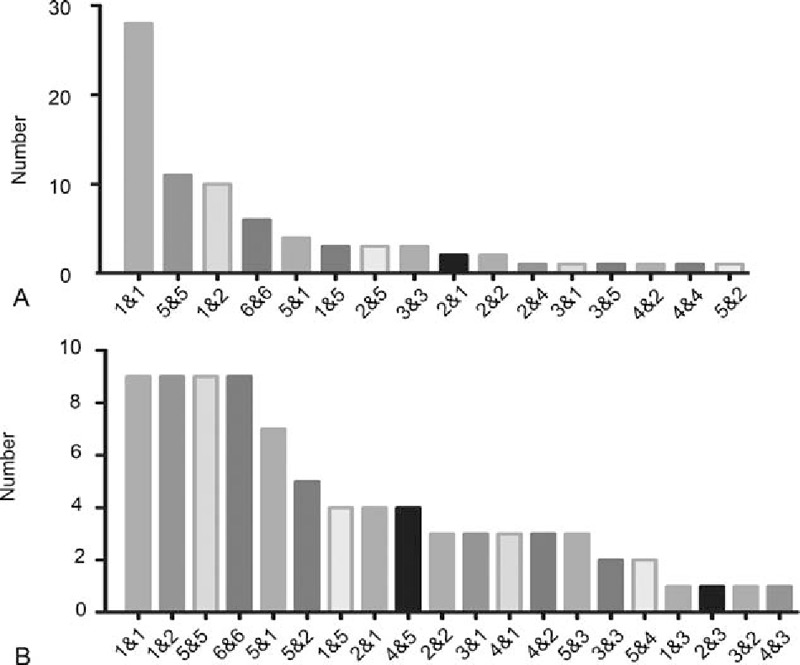
Associations among synchronous and metachronous tumors. (A) Number of patients with synchronous tumors derived from the relative systems. (B) Number of patients with metachronous tumors derived from the relative systems. 1—Digestive system, 2—respiratory system, 3—hematopoietic and lymphoid system, 4—breast tissue, 5—urogenital system, and 6—others.

In the synchronous tumor group (Fig. 1A), the most common associations were observed between the digestive and digestive system tumors (n = 28), followed by urogenital–urogenital system tumors (n = 11), and digestive–respiratory system tumors (n = 10). In the metachronous tumors group (Fig. 1B), the most frequent associations were between the digestive–digestive system tumors (n = 9), digestive–respiratory system tumors (n = 9), and urogenital–urogenital system tumors (n = 9).

More specifically, in the synchronous tumor group, the most common association was found between esophageal cancer and gastric cancer (n = 16). This was followed by associations between gastric and lung cancers (n = 5), and bladder and prostate cancers (n = 5). In the metachronous tumor group, esophageal and lung cancers (n = 5) held the first place, and esophageal and gastric cancers (n = 4) held the second place.

### 3.5 Treatment of MPMTs

Among all of the MPMT patients, 148 (92%) patients accepted therapy after the first primary cancer was diagnosed, and 145 (89.5%) patients accepted therapy after the second primary cancer was diagnosed, including surgery, chemotherapy, and radiotherapy. In the metachronous tumor group, 24 (28.9%) patients accepted therapy in terms of surgery alone, 23 (27.7%) patients accepted surgery-based comprehensive therapy (surgery combined with chemotherapy or radiotherapy), and 34 patients (41%) accepted chemotherapy or radiation therapy alone after the first primary cancer was diagnosed. In addition, 23 (27.8%) patients accepted surgery-based therapy and 39 (47%) patients did not accept surgery therapy after the second primary cancer was diagnosed. In the synchronous tumor group, 39 (50%) patients accepted the surgery therapy including the surgery-alone and surgery-based comprehensive therapy after the synchronous tumor was diagnosed, only 9 (11.5%) patients did not accept any therapy.

### 3.6 Survival outcome

**Figure 2:**
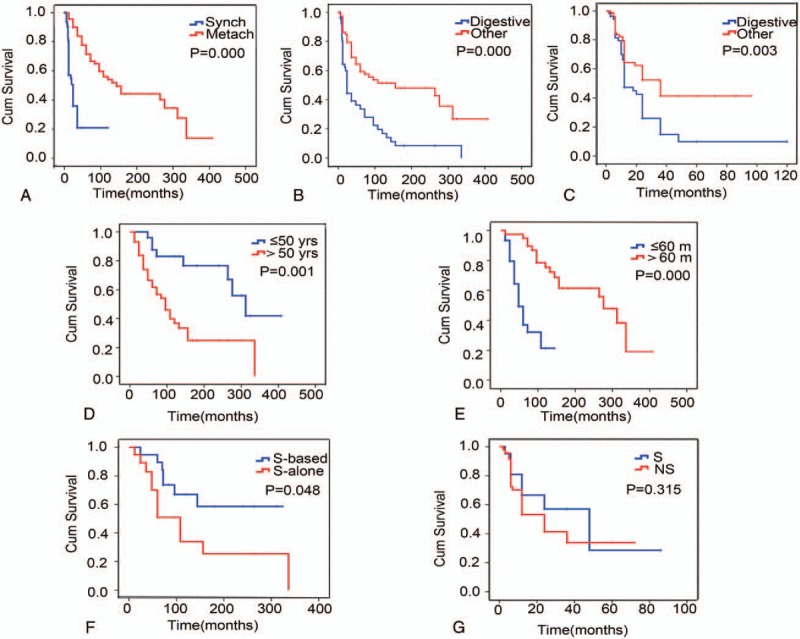
Survival times associated with different multiple primary malignant tumor groups. (A) The survival times for the synchronous or metachronous tumor groups from diagnosis of the first primary cancer. Synch: synchronous tumors, Metach: metachronous tumors. (B) The survival times for patients with digestive system tumors and tumors in other systems from diagnosis of the first primary cancer. (C) The survival times for the digestive system tumor and other systems tumor groups from diagnosis of the second primary cancer. Digestive: digestive system tumor group. Other: other systems tumor group, including the respiratory, hematopoietic, lymphoid and urogenital system, and breast. (D) The survival times of younger people (≤50 years old) and older people (>50 years old) from diagnosis of the first primary cancer in the metachronous tumor group. (E) The survival times for different interval times (≤60 vs >60 months) for the metachronous tumor group. (F) The survival times for different therapies for the metachronous tumor group from diagnosis of the first primary cancer. S-based: surgery-based comprehensive therapy, S-alone: surgical therapy alone. (G) The survival times for different therapies in the metachronous tumor group from diagnosis of the second primary cancer. S: surgical therapy; NS: no surgery.

Among the 161 patients, 138 were followed up with a median follow-up period of 36 months from diagnosis of the first primary tumor, and 78 patients died. The median survival time in the synchronous group was 12 months (range 1–120 months). The median survival time of the metachronous group was 96 months (range 12–408 months) from diagnosis of the first primary cancer and 12 months (range 2–96 months) from diagnosis of the second primary cancer. In summary, the patients with synchronous tumors showed a shorter survival time than the patients with metachronous tumors (*P* < 10^−3^) from diagnosis of the first primary cancer (Fig. 2A). In addition, for both first primary and second primary tumors, the patients with MPMTs of the digestive system showed a shorter survival time than patients with MPMTs of other systems (Fig. 2B and C).

In the synchronous group, the 1-year survival rate was 56.9%, and the 3-year survival rate was 20.9%. In the metachronous group, the survival rates differed between first and second primary cancer diagnoses; the 1-year survival rates were 95.7% and 58.0%, respectively, and the 3-year survival rates were 83.7% and 41.2%, respectively.

In the metachronous group, the survival time of younger people (≤50 years old) was longer than that of older people (>50 years old), and a short interval time (≤60 months) was associated with a shorter survival time (Fig. 2D and E). With regard to treatment, the OS time of patients differed significantly between the comprehensive therapy group (including surgery-based therapy combined with chemotherapy or radiotherapy) and the surgical therapy-alone group (*P* = .048, Fig. 2F). However, in cases of second primary cancer, there was no statistical difference in OS between the surgery-based therapy and the no surgical therapy groups (*P* = .315, Fig. 2G).

In addition, a short interval time (≤60 months) was found to be an independent poor prognostic factor of survival in the metachronous cancer group based on Cox regression hazard model (Table 5). Specifically, a short interval (≤60 months) was associated with a shorter survival time. However, in the synchronous group, there were no detectable differences regarding age, gender, or treatment types based on either Kaplan–Meier or Cox proportional hazard multivariate analyses.

**Table 5:**
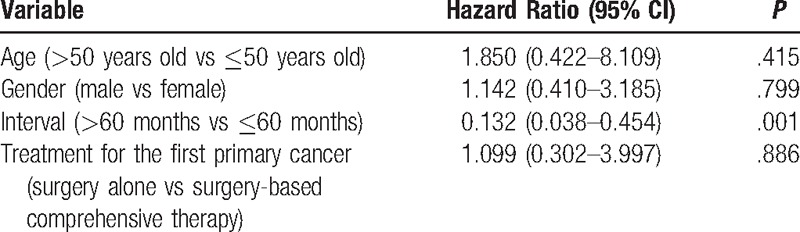
Analysis of prognostic factors contributing to mortality of metachronous MPMTs.

## 4 Discussion

MPMT is a special phenomenon in tumorigenesis that is beginning to become better understood due to a number of studies worldwide. Studies have reported that the incidence of MPMTs was 0.52% to 3.66% in China compared with 0.73% to 11.7% in other countries.^[8–10]^ In our hospital in Shaanxi, China, the incidence of MPMTs was 1.0%, which is similar to the rates of prevalence previously reported in China but less than those reported in other countries. Many factors may contribute to the diverse incidence of MPMTs in different geographical regions, such as genetic factors, environmental factors, diagnostic methods, and follow-up information.

The prevalence of synchronous MPMTs has differed between previous studies (range 30%–55%).^[3,11,12]^ In our study, 48.4% of patients had synchronous type and 52.6% of the patients had metachronous type tumors. This might be a result of the different population characteristics, different diagnostic tools, and different rules of cancer registry between hospitals. In our study, the prevalence of synchronous and metachronous MPMTs was similar, emphasizing the importance of an accurate synchronous diagnosis.

MPMTs can occur at any age. However, earlier studies reported that patients with MPMTs tend to be older than those with a single primary cancer, and in most reports, more than 75% of patients with MPMTs were more than 50 years of age.^[13–15]^ Our results were consistent with these earlier studies, with 77.6% of patients being over 50 years old. However, the median age of diagnosis of the first primary cancer was lower in the metachronous group than in the synchronous group, indicating that young people are more likely to have metachronous tumors and should be closely monitored for a longer time.

As reported by some previous studies,^[16,17]^ our results showed a short interval time (≤60 months) was associated with a shorter survival time. This may indicate that the first primary cancer had been cured over a longer interval time. The interval for more than 50% of patients was within 60 months, indicating that screening for second primary cancers should be performed within 5 years emphatically. However, our results showed that among the 22 patients with an interval time of more than 120 months, the first primary cancers of 12 of these patients were breast and urogenital system cancers. This may reflect the long survival time of breast and urogenital system cancers, and these patients should therefore be monitored beyond 60 months.

Consistent with other reports,^[18,19]^ men were more frequent than women among both the synchronous and metachronous groups in our study. In addition, more than 60% of patients with MPMTs exhibited primary tumors of the digestive and respiratory systems in men. The main reasons for the high incidence of tumors of the digestive and respiratory systems were tobacco and alcohol in men.^[20,21]^ This indicated that these 2 sites should be closely monitored by screening, especially in men. Men should also be advised of the risk between smoking and alcohol consumption and developing MPMTs or a single primary cancer.

In our study, the most frequent sites of localization and pathology of tumors were the digestive system and adenocarcinomas, respectively. Within the digestive system, the stomach and the esophagus were the most frequent sites of tumor localization. This was not in agreement with previous reports.^[3,9,22–25]^ We think that the main reason for this difference may be regional differences. In developed countries, the most frequent site of tumor localization was the colorectum.^[22]^ In Japan, the incidence of gastric cancer with colorectal cancer was reported to be high.^[23]^ In Turkey, the most frequent site of MPMT localization was the skin.^[3]^ In Guang Dong province, China, nasopharyngeal carcinoma was the most frequent reported for MPMTs.^[9]^ In Beijing, the most frequent site for MPMTs was breast tissue.^[24]^ However, in Shaan Xi province, the most frequent site of tumor localization was the upper gastrointestinal tract, including gastric and esophageal cancers.^[25]^ This demonstrated that in less-developed regions, MPMTs were focused predominantly within the upper gastrointestinal tract of patients, likely related to economic factors such as poor eating habits.

When analyzed in more detail, MPMTs were most frequently associated with the stomach and the esophagus (synchronous group, n = 16; metachronous group, n = 4) in our study. There are a number of possible explanations for this. First, studies have reported many similar genetic changes between gastric and esophageal cancers. Hence, these 2 types of cancer potentially share similar molecular mechanisms for pathogenesis.^[26,27]^ Second, the stomach and the esophagus are both part of the digestive system and would therefore potentially be exposed to the same pathogenic factors.^[28,29]^ Third, gastric and esophageal cancers can be detected simultaneously by endoscopy. In less-developed regions, it may therefore be prudent to focus on the detection of gastric and esophageal cancers by endoscopy at follow-up.

Some studies reported about the MPMTs with soft tissue sarcoma (STS) and GIST. Of STS patients, 6% to 8% developed MPMTs, which were most frequently associated with STS and breast carcinoma and genitourinary malignancies. Physicians should be aware of patients with primary malignant fibrous histiocytoma who demonstrate a risk for developing a renal cell carcinoma.^[30–32]^ Of the GIST patients, 10% to 20% developed MPMTs. The most common association with GIST was the stomach, the prostate, the breast, the esophagus, and the kidney cancer. The report indicated that the patients with GIST with 2 or more other cancers had a poor prognosis.^[33,34]^ So these patients with GIST and other cancers should be noted. In addition to the frequent MPMTs, there are rare cases, such as giant cavernous hepatic hemangioma with endometrial adenocarcinoma, synchronous adrenocortical carcinoma with GIST, synchronous jejunal carcinoid tumor with colorectal polyps, and bilateral synchronous sporadic renal cell carcinoma.^[35–38]^ Because of the rarity of the incidence of these MPMTs, we need more data to explore their association.

The high risk for MPMT patients who did not have a family history of malignancy might be in part due to the environmental factors and/or genetic factors that are shared between the 2 malignancies. The specific mechanism of MPMTs is not unclear until now. Recently, some studies reported that many genes, including *BRCA2*, *ATM*, *POLD1*, *PABL2*, *SMAD4*, and so on, played an important role in the occurrence and pathogenesis of MPMTs.^[39–41]^ Specifically, the germline mutations of BRCA1/BRCA2 were associated with increased risk of breast, ovarian, stomach, colorectum, uterus, and pancreas cancers. ATM truncations were also detected in many cancer types, mostly in lung, stomach, and prostate cancers.^[39]^*PABL2* gene variation was associated with increased risk of ovarian and stomach carcinoma.^[39]^ The POLD1 mutation was also associated with colorectal cancer and endometrial cancer predisposition.^[41]^ More and more studies reported the common gene variations in different types of cancers.

In addition to the gene list, significant associations have been previously noted between the microsatellite instability (MSI) phenotype and multiple primary malignancies. Genetic instability may play an important role in the development of second primary tumors. Therefore, testing for MSI in the primary cancer might help detect those patients who are at high risk for developing double primary malignancies.^[42–44]^

The high risk of MPMTs is also associated with the ways and effects of treatment.^[45]^ In the synchronous tumor group, 50% patients accepted the surgery therapy after the synchronous tumor was diagnosed. But the treatment strategies for synchronous and single tumors are different. With the example of colorectal cancer, some authors have suggested that total or subtotal colectomy should be performed.^[46,47]^ Passman et al^[48]^ recommended a more extensive resection for lesions in adjacent segments. Lee et al^[49]^ suggested that 2 regional resections are preferable through the comparison between the 2 regional resections and extensive resection approaches. Therefore, there has been little agreement among surgeons regarding the appropriate surgical treatment for synchronous cancers located in separate segments. This need more study to answer the question.

Moreover, in our study, the patients who accepted surgery-based comprehensive therapy (surgery combined with chemotherapy or radiotherapy) had a longer survival time than the patients who accepted surgery alone. These results indicate that doctors should carefully design treatment strategies to include chemotherapy or radiation therapy according to current guidelines. In addition, there was no statistically significant difference in the OS time from diagnosis of a second primary cancer between the surgery-based therapy and the no surgery groups after the second primary group was diagnosed. Therefore, doctors should perform a careful preoperative evaluation to determine whether there is a need for surgery.

Our findings also showed that patients with synchronous tumors displayed shorter survival times than patients with metachronous tumors (*P* ≤ .000) from diagnosis of the first primary cancer. In all MPMT cases, either for the first primary or the second primary tumors, patients with MPMTs of the digestive system showed shorter survival times than patients with MPMTs of other systems. This difference may be due to the characteristics of metachronous and digestive system tumors. In addition, in the metachronous group, the survival rate of younger people (≤50 years old) was higher than that of older people (>50 years old). The reasons for this may include the following: first, older patients have an increased incidence of cardiac and cerebral vascular problems; second, older patients have had more time to potentially accumulate pathogenic genes; third, the life expectancy of young people is longer; and lastly, treatment strategies in different ages of patients are different.

However, our study has some limitations. Comprehensive analysis of MPMTs requires the observation of a large, well defined population for ≥10 years. We studied only partial data from different departments within a single hospital, and the median follow-up period was only 36 months. Furthermore, we did not consider the staging of tumors, environmental factors such as smoking and diet, radiation fields, or other complications such as cardiac and cerebral vascular incidents.

## 5 Conclusion

Our study showed that patients with digestive, urogenital, and respiratory tumors were more likely to develop MPMTs. In particular, older people (>50 years old) and men were high-risk populations. Short interval times (≤60 months) were associated with a poor prognosis for patients with metachronous cancer. Therefore, careful surveillance and follow-up are especially important in these patients.

## Acknowledgments

Statistical analysis involved in this article was reviewed by statistician Min Yi, Department of Breast Surgical Oncology, The University of Texas MD Anderson Cancer Center, Houston, TX.

The authors thank the patients and family members for their participation in this study.
